# A Cohort Study of Myocardial Perfusion Imaging in Veteran Patients Without Symptoms: Contributing Factors and Results of Testing

**DOI:** 10.1097/MD.0000000000001154

**Published:** 2015-08-14

**Authors:** David E. Winchester, Pengcheng Zhang, Manoj P. Jadhav, Rebecca J. Beyth

**Affiliations:** From the Malcom Randall Veterans Affairs Medical Center, Gainesville, Florida, USA (DEW, RJB); Division of Cardiovascular Medicine, Department of Medicine, College of Medicine, University of Florida, Gainesville, Florida, USA (DEW, MPJ); College of Medicine, University of Florida, Gainesville, Florida, USA (PZ); and Division of General Internal Medicine, Department of Medicine (RB), College of Medicine, University of Florida, Gainesville, Florida, USA (RJB).

## Abstract

Myocardial perfusion imaging (MPI) is commonly used to detect ischemia. Concerns about silent ischemia may encourage orders for MPI in asymptomatic patients. Factors contributing to this practice are poorly described and the clinical utility is questionable.

We conducted a single center retrospective cohort investigation on Veterans who underwent MPI between December 2010 and July 2011. We gathered data on symptoms, baseline characteristics, results of MPI, and cardiovascular events within 1 year. MPI were categorized using 2009 appropriate use criteria (AUC).

Of 592 patients, 127 (21.5%) had no symptoms at the time of MPI. Comparing symptomatic and asymptomatic patients, no differences were observed in baseline characteristics except abnormal ECG, more common in asymptomatic patients (n = 86, 67.7% vs. n = 232, 49.9% for symptomatic patients, *P* < 0.0001). Asymptomatic MPI were more commonly inappropriate (n = 26, 21.5% vs. n = 31, 6.7% for appropriate/uncertain, *P* < 0.0001). Detection of ischemia between patients with and without symptoms was not different (*P* = 0.86); however, among asymptomatic MPI that also demonstrated ischemia, none were inappropriate (n = 10 appropriate, n = 7 uncertain). In multivariate regression, 2 factors were associated with asymptomatic status, abnormal ECG (odds ratio [OR] 2.29, 95% confidence interval [CI] 1.5–3.49) and age over the median (OR 0.63, 95% CI: 0.41–0.95).

A substantial portion of MPI tests are ordered for patients without symptoms. When compared to symptomatic patients, MPI for asymptomatic patient were more commonly inappropriate; however, the prevalence of ischemia was similar. MPI may be clinically relevant in some asymptomatic patients and decisions to test should be based on the AUC.

## INTRODUCTION

In clinical medicine, diagnostic tests are ordered for a variety of reasons such as screening, evaluating symptoms, and assessing for risk. With regard to coronary artery disease (CAD), myriad tests are available for these purposes. In some cases, such as nuclear myocardial perfusion imaging (MPI), one modality can be used all the above situations. Because CAD is one of the most common causes of death in the world, doctors and patients share enthusiasm for early detection and prevention.

Widespread screening for coronary ischemia with nuclear MPI would be impractical, costly, and risky (given the associated radiation). In selected patients at high risk of cardiovascular events, however, the risks and costs may be worthwhile if the strategy sufficiently improved outcomes. Early enthusiasm for this approach^1,2^ has been tempered by recent randomized trial data which suggest no net benefit even in high risk populations.^3^

To inform clinicians which patients are likely to benefit from MPI, professional societies related to cardiology and medical imaging have developed appropriate use criteria (AUC).^4^ Based on the available evidence, the AUC recognize that MPI is rarely appropriate for patients with no symptoms or stable symptoms. In a prior publication, we demonstrated that approximately 10% of nuclear MPI at our institution are inappropriate,^5^ similar to reports from other facilities.^6^ We also observed a substantial proportion of patients underwent MPI despite having no symptoms at the time. We conducted the current investigation to assess the clinical utility of MPI in patients without symptoms. We hypothesized that MPI performed in asymptomatic patients would detect ischemia less frequently than MPI done for patients with symptoms.

## METHODS

### Patient Study Groups and Data Collection

We conducted a retrospective cohort study on Veterans who underwent MPI studies at 1 of 2 nuclear cardiology laboratories within the North Florida/South Georgia Veterans Affairs Medical Center in Gainesville, FL. Consecutive patients who underwent MPI between December 2010 and July 2011 were included and were divided into 2 cohorts, asymptomatic and symptomatic. MPI were performed using either rubidium-82 positron emission tomography (PET) or technetium-99m single photon emission computed tomography (SPECT) combined with either treadmill exercise stress or regadenoson vasodilation. MPI could be ordered by any physician or advanced provider within our facilities, regardless of their area of clinical practice; the majority were ordered by primary care providers in the outpatient setting. Relevant data regarding patient baseline demographic, clinical characteristics, and results of MPI testing were collected from the Computerized Patient Record System. The reason for ordering MPI tests was determined from patient history of medical progress notes and documentation from providers who ordered the test. Diabetes mellitus was defined as hemoglobin A1C greater than 8% or the documented prescription of oral/injected medications for treatment of diabetes. CAD was defined as prior myocardial infarction (MI) or revascularization. Data on patient cardiac events were gathered for up to 1 year after the index MPI was performed. Data regarding the MPI results (test conclusion, summed stress score, and summed difference score) were taken from the final official report of the test; we did not reinterpret the images. Our Institutional Review Board reviewed this study and waived the requirement for written informed consent.

### Appropriateness Categorization

Appropriateness indication for subjects was categorized retrospectively using a custom paper form based on the Imaging in FOCUS (Formation of Optimal Cardiovascular Utilization Strategies) initiative.^7^ Appropriateness categorization was performed using the 2009 AUC by acknowledged coinvestigators (RM, DN, SR, RC). Data collection occurred in 2012, before publication of the current iteration of AUC for stable ischemia heart disease.^8^ We used the standard terms for categorizing MPI: appropriate, uncertain, and inappropriate. Extensive description of the meaning behind these terms is provided in the published AUC documents. No formal interrater or intrarater assessments of consistency were performed. In the event of discrepancies, the primary author (DEW) finalized the categorization.

### Outcome and Statistical Analysis

The primary outcome of this study was to determine if the prevalence of ischemia (defined as a summed difference score greater than 5) was less common in asymptomatic patients, as compared to symptomatic patients. Using our established cohort of 592 patients, we estimated that we would have power (1-beta) of 78% to detect a 10% difference in the prevalence of ischemia (2-tailed, alpha = 0.05). Power analysis was performed using G∗Power 3.^9^ Categorical variables including the prevalence of ischemia patient characteristics, and additional cardiac events were compared between the symptomatic and asymptomatic population by *t* test and Chi-square tests as appropriate. Additional cardiac events included MI, subsequent catheterization, revascularization, and death. We developed a multivariate logistic regression model using a forward conditional stepwise approach to determine associations between patient characteristics and symptom status; *P*-value < 0.05 was required to be retained in the model. Variables included in this model were: sex, diabetes, hypertension, abnormal electrocardiogram (ECG), CAD, obesity, current smoking, and age above the median (63 years). Statistical analysis was performed using SPSS version 21 (IBM; Armonk, NY). We applied the Strengthening the Reporting of Observational Studies in Epidemiology method in design of our investigation.^10^

## RESULTS

### Baseline Characteristics

Of 592 patients who underwent MPI, 88.5% were symptomatic (N = 465), and 21.5% (N = 127) were asymptomatic at time of MPI. The population was overwhelmingly male with a high prevalence of obesity, hypertension, and hyperlipidemia (Table 1). An abnormal ECG was the only baseline patient characteristic different between the 2 cohorts (symptomatic 49.9% vs. asymptomatic 67.7%, *P* < 0.0001). PET was performed on the majority of patients (90%); no differences in baseline characteristics were observed between the PET and SPECT patients. In the symptomatic cohort, the following were reported (patients could report multiple symptoms): chest pain 62.8%, dyspnea 51.6%, fatigue 12.5%. Within those with chest pain, 1.5% were typical, 11.0% were atypical, and 87.5% were noncardiac.

**TABLE 1:**
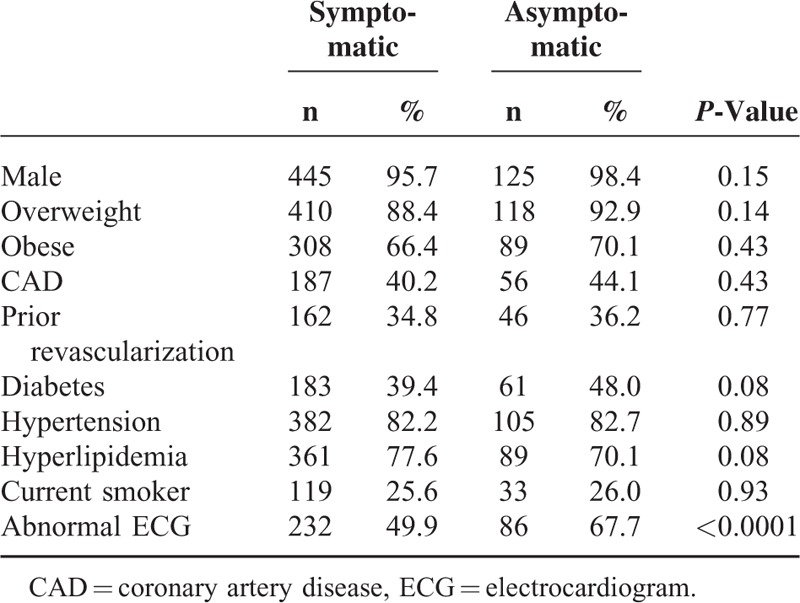
Baseline Characteristics

**FIGURE 1:**
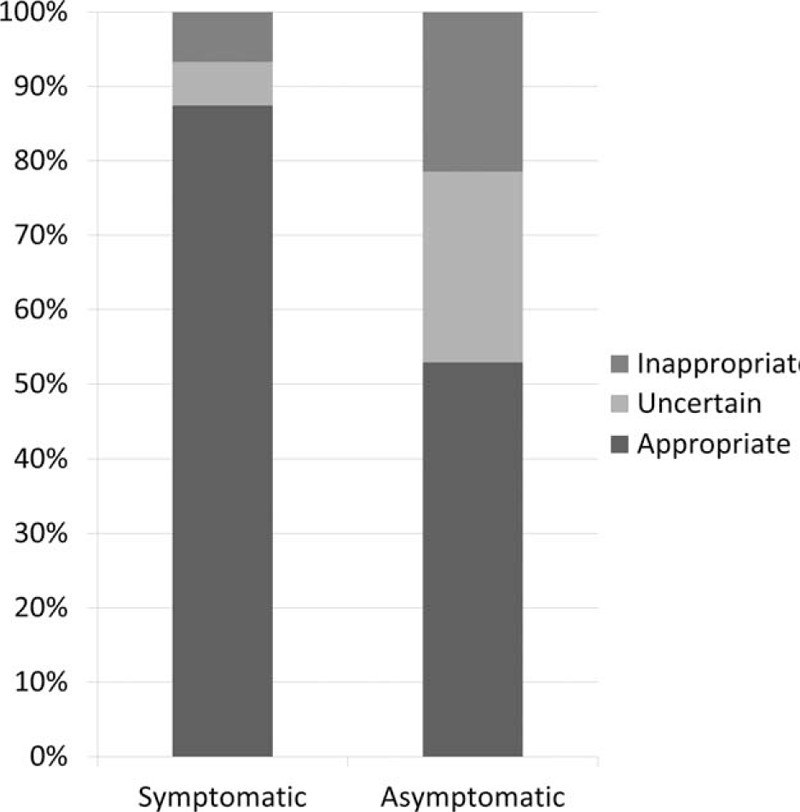
Distribution of appropriateness between symptomatic and asymptomatic patients. This stacked bar graph displays the proportion of nuclear test appropriateness ratings. Approximately half (52.8%) of nuclear tests for patients without symptoms were rated as appropriate, compared to 86.7% for those with symptoms.

Among MPI performed in the symptomatic population, 86.7% (N = 403) were appropriate, 5.8% (N = 27) uncertain, and 6.7% (N = 31) inappropriate; for 4 patients, appropriateness category could not be determined (Figure 1). In the asymptomatic population, 52.8% (N = 64) were appropriate, 25.6% (N = 31) uncertain, and 21.5% (N = 26) inappropriate. Comparing the distribution of appropriateness between cohorts, asymptomatic patients were more likely to be inappropriate (21.5% vs 6.7%, *P* < 0.0001).

Multivariate regression was performed for further analysis of correlation between baseline clinical characteristics and symptom status (Table 2). An abnormal ECG was associated with being asymptomatic (OR = 2.29, 95% CI: 1.5–3.49) while age over the median was associated with being symptomatic (OR = 0.63, 95% CI: 0.41–0.95).

**TABLE 2:**
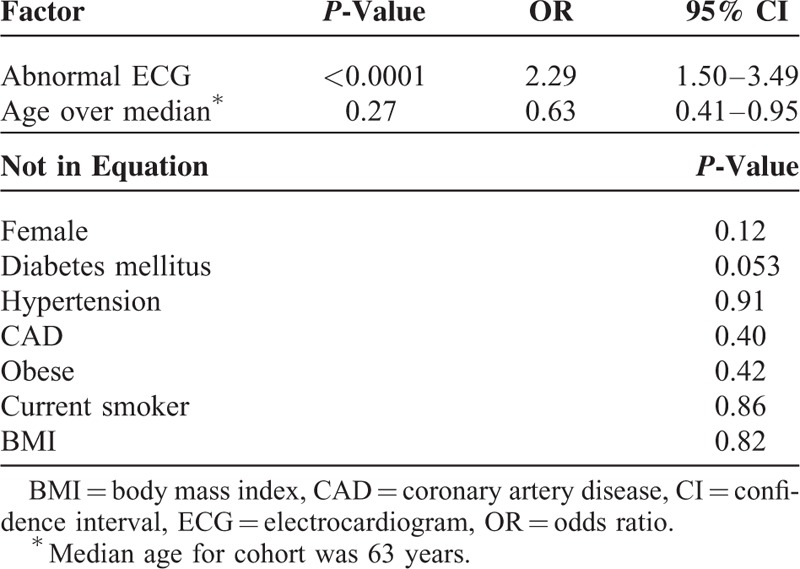
Multivariate Regression of Factors Associated With the Absence of Symptoms

### Outcomes

The primary outcome, the prevalence of ischemia on MPI, was not different between the symptomatic and asymptomatic cohorts (13.6% vs. 14.3%, *P* = 0.86) (Table 3). No differences were observed between the asymptomatic and symptomatic populations for the other cardiovascular outcomes including MI (*P* = 0.644), catheterization (*P* = 0.562), revascularization (*P* = 0.762), and death (*P* = 0.376). Among asymptomatic patients with abnormal MPI, none of the ordered studies were inappropriate (N = 10 appropriate, N = 7 uncertain).

**TABLE 3:**
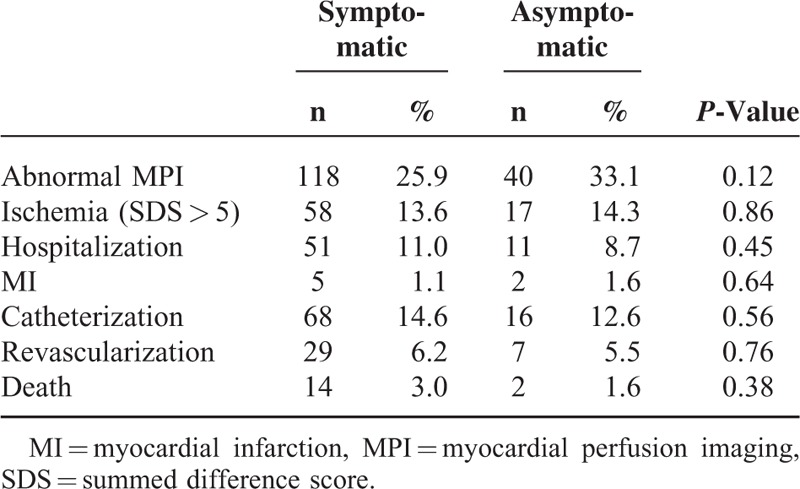
Results of MPI and Cardiovascular Events Within 1 Year

## DISCUSSION

On first impression, nuclear MPI testing for patients without symptoms may seem to be an inappropriate use of the technology. In our investigation of this phenomenon, we observed that MPI for asymptomatic patients was frequently inappropriate but not universally so. A substantial portion of MPIs in asymptomatic patients were abnormal; none of which were rated as inappropriate based on the 2009 AUC for MPI. In multivariate regression, patients undergoing MPI while asymptomatic were more likely to be younger or have an abnormal ECG. The prevalence of nonspecific symptoms, such as dyspnea, increases with age, which may explain the age component of our regression analysis.^11^ Anecdotally, many providers at our facility consider that an abnormal ECG warrants screening for CAD. Use of MPI in younger patients is of potential concern given the nontrivial exposure to ionizing radiation during MPI.

The clinical relevance of our findings is that MPI in asymptomatic patients should not be immediately dismissed as inappropriate, but should be considered in the context of a more nuanced evaluation of appropriateness. The AUC provide the nuance necessary to distinguish between appropriate and inappropriate tests and we encourage clinicians to use them in making clinical decisions about testing. Examples from the AUC where MPI for asymptomatic patients is appropriate include preoperative assessment for high risk surgery or for patients who cannot provide an estimate of exertional capacity (2009 AUC indications #43 and #47). Another example would be patients with a recent acute coronary syndrome who do not undergo invasive coronary angiography (2009 AUC #50 and #52) or CAD patients without complete revascularization (2009 AUC #56). In the 2009 AUC, asymptomatic patients with high coronary heart disease risk were considered appropriate for MPI (#15) although this rating was downgraded in the 2013 AUC (#10), informed by data from the DIAD trial.^3,8^ Aside from a few appropriate indications for MPI in asymptomatic patients, the AUC generally discourage the practice. In the 2009 version, out of 67 indications, 34 relate to patients with stable or no symptoms, of which 9 were rated appropriate (9 of 67, 13%). In the 2013 version (for stable heart disease only), 37 of 80 total indications relate to stable/asymptomatic patients with only 4 rated as appropriate (4 of 80, 5%).

Despite these recommendations, the use of MPI in patients without symptoms appears to be a common contemporary practice. A recent information statement from the American Society of Nuclear Cardiology details indications where testing in asymptomatic individuals may be fruitful, along with a summary of the strength of evidence for those indications.^12^ Other large studies on the appropriate use of MPI have reported similar or higher proportions of asymptomatic patients as well as a strong correlation between lack of symptoms and inappropriate rating for MPI.^13,14^ The reasons for this practice pattern are difficult to ascertain, however is likely driven at least partially by concerns about silent ischemia in both patients with and without known CAD.^15^ Evidence for cardiovascular risk with silent ischemia has been described for decades with both ambulatory ECG monitoring and MPI.^16,17^ Studies such as the Asymptomatic Cardiac Ischemia Pilot study demonstrated that the risk from silent ischemia could be mitigated by revascularization.^18^ Clinical Outcomes Utilizing Revascularization and Aggressive Drug Evaluation (COURAGE) nuclear substudy showed us that patients with successful reductions in ischemia suffered fewer cardiovascular events.^19^ The overall population in the COURAGE trial, however, had similar outcomes for revascularized and medically treated patients with stable CAD.^20^ As such, patients with high cardiovascular risk and those with silent ischemia can likely be adequately managed with medical therapy alone and will not benefit from MPI. As such, the decision in the 2013 AUC to downgrade many of the asymptomatic indications for MPI seems prudent.

## LIMITATIONS

Our data were collected using the 2009 AUC. The indications for stable ischemic heart disease have recently been updated with significant changes to the indications for MPI in asymptomatic patients. More contemporary data sets may have contrary findings. This investigation was conducted in a single academically affiliated Veterans Affairs medical center; the population is different from a general medical population and may not be applicable. The investigation was not adequately powered to detect differences in cardiovascular outcomes.

## CONCLUSION

A substantial portion of MPI tests are ordered for patients without symptoms. Compared to symptomatic patients, MPI for asymptomatic patients were more commonly inappropriate; however, the prevalence of ischemia was similar between the groups. As outlined in the AUC, some populations may warrant MPI despite a lack of symptoms.
